# Rituximab for Refractory Rheumatoid Arthritis: A 24-Week Open-Label Prospective Study

**DOI:** 10.2174/1874312900701010001

**Published:** 2007-09-18

**Authors:** Ling Yin Ho, Chi Chiu Mok, Chi Hung To, Anselm Mak, Mei Yuk Cheung, Ka Lung Yu

**Affiliations:** Department of Medicine and Geriatrics, Tuen Mun Hospital, Hong Kong SAR, China

**Keywords:** Biologics, rituximab, anti-TNFα, refractory, treatment.

## Abstract

**Objectives:**

To study the efficacy of rituximab in active rheumatoid arthritis (RA) patients refractory to disease modifying anti-rheumatic drugs (DMARDs) including the tumor necrosis factor (TNF)-α antagonists.

**Methods:**

Adult patients with active RA despite adequate therapies with conventional DMARDs or anti-TNFα agents for at least 3 months were recruited. Inclusion criteria were: (1) Positive RF / anti-CCP; (2) ≥ 6 swollen joints and ≥ 8 tender joints; (3) ESR ≥ 28 mm/hr or CRP ≥ 10 mg/L. Eligible patients were given intravenous rituximab infusions at a dose of 1000 mg on days 1 and 15. Assessment was performed 4-weekly thereafter and included tender joint counts (TJC), swollen joint counts (SJC), physician’s and patient’s global assessment, patient’s pain assessment (VAS 0-100 mm), disability index (HAQ-DI), quality of life (SF36), fatigue score (FACIT-F), ESR and CRP. The DAS28, EULAR and ACR responses at week 24 were evaluated.

**Results:**

10 patients (8 women and 2 men) were studied (mean age: 49 years; mean RA duration 7.4 years). Baseline TJC and SJC were 25.1 ± 13.2 and 12.8 ± 5.4 respectively. The mean DAS28 score was 7.1 ± 0.7, and the mean CRP and ESR levels were 52.3 ± 60 mg/L and 95.8 ± 32 mm/hr, respectively. The median number of failed DMARDs was 4 and two patients had failed anti-TNFα treatment. At week 24, there was a significant drop in TJC, SJC, ESR and CRP. The HAQ-DI score also decreased from 2.1 to 1.7 (p=0.04) while the total SF-36 score improved from 24.8 to 38.3 (p=0.008). Sixty percent of patients achieved EULAR moderate-to-good response. Half of the patients achieved ACR20 and two achieved ACR50 / 70 response. Only one patient experienced a minor infusion reaction.

**Conclusions:**

Rituximab is effective and well tolerated in patients with refractory RA.

## INTRODUCTION

Rheumatoid arthritis (RA) is a chronic inflammatory disease that leads to significant disability and mortality. Conventional disease modifying anti-rheumatic drugs (DMARDs) include methotrexate (MTX), leflunomide (LEF), sulphasalazine (SSz), hydroxychloroquine (HCQ) and gold compounds. Despite the use of these agents as monotherapy or combination therapy, a substantial proportion of patients still cannot achieve a clinically meaningful clinical response. For patients with inadequate response to conventional DMARD therapies, the anti-tumor necrosis factor (TNF)-α agents such as infliximab, etanercept and adalimumab are treatment options. However, data from western countries have shown that around 50-60% of patients may achieve an American College of Rheumatology (ACR) 20 response [1-3]. Other patients are either withdrawn from the TNFα inhibitors because of inefficacy or experiencing adverse effects.

Because of the lack of universal efficacy of the TNFα inhibitors, a number of non-TNFα biological agents have been developed. One of these, rituximab, is a chimeric monoclonal antibody directed against the CD20 antigen on the surface of B cells [4]. Administration of rituximab leads to selective depletion of the CD20 positive B cells from the body, leaving the stem cells and plasma cells unaffected because CD20 is not expressed on these cells.

Rituximab was first reported to be useful in refractory RA [5]. A phase II randomized controlled trial has shown efficacy in RA patients refractory to MTX [6]. With the addition of rituximab, an ACR20 response could be achieved in 73% of the patients as compared to 38% when MTX alone was continued. An extended follow-up of these patients at 48 weeks showed that the ACR response was sustained in some patients, with significant improvement in physical function, as measured by the Health Assessment Questionnaire Disability Index (HAQ-DI) being evident at week 24 [7]. A further extended follow-up of these patients at 1 and 2 years showed that a significantly higher proportion of patients who received rituximab plus MTX had improvement in HAQ-DI to a greater extent than the minimally clinically significant difference than those who received MTX alone [8].

A more recent double-blind placebo-controlled clinical study (DANCER) confirmed that in RA patients who were refractory to conventional therapies and the TNFα inhibitors, rituximab treatment (2 doses of 1000 mg or 500 mg) led to significantly higher rates of ACR responses and EULAR moderate / good responses when compared to placebo infusion [9]. Rituximab was safe and well tolerated. The frequency of infectious complications was similar between treatment and placebo groups. Reactions to the first infusion occurred in one-third of patients receiving 1000 mg rituximab infusion and could be minimized by intravenous corticosteroid pre-medication.

The experience of rituximab in RA is limited in Hong Kong. We conducted this open-label cohort study to evaluate the efficacy and tolerability of rituximab in our local Chinese patients with active RA who were refractory to DMARD and anti-TNFα therapies.

## PATIENTS AND METHODS

Patients with active RA despite conventional DMARD or anti-TNFα therapies were recruited. The inclusion criteria were: (1) ≥ 18 years of age; (2) 1987 ACR criteria for the classification of RA [10]; (3) Ability to give informed consent and comply with the protocol and assessment; (4) Positive for either rheumatoid factor (RF) or anti-CCP antibody; (5) Active RA despite therapies with conventional DMARDs or the anti-TNF agents for at least 3 months, as evidenced by ≥ 6 swollen joints (66 joint count system) and ≥ 8 tender joints (68 joint count system); (6) Raised erythrocyte sedimentation rate (ESR) to ≥ 28 mm/hr or C-reactive protein (CRP) ≥ 10 mg/L; (7) Stable dose of conventional DMARDs for at least 8 weeks prior to study entry or washout for at least 4 weeks (except for methotrexate and leflunomide, which were to be continued); (9) Use of anti-TNFα agents (infliximab, etanercept or adalimumab) for at least 3 months and washout (etanercept for at least 2 weeks, infliximab or adalimumab for at least 8 weeks).

Exclusion criteria were: (1) Major surgery (including joint surgery) within 8 weeks prior to study entry; (2) Functional class IV as defined by the ACR classification of functional status in RA [11]; (3) Treatment with other investigational agents within 4 weeks of study entry (eg. anti-IL6, anti-CD4); (4) Treatment with gamma globulin, plasmapheresis or Prosorba column within six months of study entry; (5) Intra-articular steroid injection 6 weeks before study entry; (6) Immunization with a live/attenuated vaccine within 4 weeks prior to study entry; (7) History of severe allergic reaction to human, humanized or murine monoclonal antibodies; (8) Active current bacterial, viral, fungal, mycobacterial or other infections; (9) Chronic hepatitis B or hepatitis C carriers; (10) History of malignancies, including solid tumors and hematologic malignancies; (11) Pregnant women or lactating mothers.

This study was approved by the Research and Ethics committee of our hospital. Written consent was obtained from all participants.

### Protocol

Patients were given rituximab 1000 mg by intravenous infusion on Day 1 and Day 15. Routine pre-medication was not given. Intravenous hydrocortisone (100 mg) and chlorpheniramine (10 mg) were to be given if patients developed a reaction to the infusion. Methotrexate was to be continued throughout the study period, together with folic acid. Leflunomide was also to be continued. The use of non-steroidal anti-inflammatory drugs (NSAIDs) was allowed. Other DMARDs were stopped for at least 4 weeks prior to study entry.

### Clinical Assessments on Follow-Up Visits

Patients were followed up four-weekly for clinical response and side effects to rituximab infusion. The following was assessed: tender joint count ([TJC]; 0-68), swollen joint count ([SJC]; 0-66), patient’s pain assessment, patient’s global assessment and physician’s global assessment as recorded by visual analog scale (VAS) 0-100 mm. Physical functioning and disability was assessed by Health Assessment Questionnaire-Disability Index (HAQ-DI). Quality of life was assessed by the short form (SF)-36 questionnaire (Hong Kong Chinese version) [12]. Fatigue was assessed by the Functional Assessment of Chronic Illness Therapy-Fatigue (FACIT-F) subscale [13]. The DAS28 scores were calculated at each clinic visit.

### Study End Points

The primary end-point of the trial was the proportion of patients who met the ACR20 and EULAR response criteria.

### Adverse Events

A checklist of possible adverse events related to rituximab was evaluated for participants at each clinic visit.

### Statistical Analyses

Unless otherwise stated, values expressed were mean ± standard deviation (SD). Continuous variables from baseline to various time intervals were compared by the non-parametric Wilcoxon signed rank test. Statistical significance was defined as a p value of less than 0.05, two-tailed. Statistical analyses were performed by the SPSS program (version 11.5, Chicago IL, 2002).

## RESULTS

Twelve patients were screened but two of them were not eligible for study entry because of inadequate number of swollen and tender joints. Ten patients (two men and eight women) were finally recruited. All of them were ethnic Chinese. The median age was 49 years (range 42 to 62). The demographic and baseline disease characteristics are shown in Table **1**. At study entry, all patients had very active RA as evidenced by a large number of TJC (25.1 ± 13.2) and SJC (12.8 ± 5.4), elevated ESR (95.8 ± 32.3 mm/hr) and CRP (52.3 ± 53.5 mg/L) levels. Seven patients had radiological erosions at baseline. The median number of ineffective DMARDs in these patients was four. Two patients had failed anti-TNFα treatment (infliximab in one and both etanercept and infliximab in another patient).

Figs. (**1**,**2**) show the number of patients who achieved the EULAR and ACR responses, respectively, at baseline and different time intervals. At week 24, 60% of the patients achieved EULAR moderate-to-good response. Half of the patients achieved ACR20, one achieved ACR50 and one achieved ACR70 response. The mean DAS28 scores decreased significantly from 7.1±0.7 at baseline to 5.6±1.2 at week 24 (p=0.005). Of the two patients who had failed anti-TNFα treatment, one showed moderate EULAR response while the other did not meet the criteria for a clinical response despite a numerical improvement in the joint counts and DAS score.

Comparing the data at week 24 with baseline, there was a significant drop in the mean number of TJC (25.1 ± 13.2 to 13.9 ± 14.7, p=0.005), and SJC (12.8 ± 5.4 to 5.7 ± 5.2, p=0.02). This was accompanied by a significant improvement in the mean levels of ESR (95.8 ± 32.2 to 65.4 ± 41 mm/hr, p=0.01) and CRP (52.3 ± 53.5 to 30.1 ± 42.6 mg/L, p=0.02).

The mean HAQ-DI score decreased from 2.1 ± 0.4 to 1.7 ± 2.6 (p=0.04). Seventy percent of patients had an improvement from baseline of greater than 0.25 points of the HAQ-DI, ie. the minimum clinically important difference (MCID) as described by Strand *et al*. [8]. Both the mental and physical health sub-scores of the SF-36 increased significantly from baseline to week 24. The mean fatigue (FACIT-F) score also improved significantly from 27.7 ± 7.4 to 20.1 ± 6.6 (p=0.007).

Finally, the mean titers of RF dropped significantly from baseline to week 24 (42.0 ± 14 to 30.4 ± 12 IU/ml; p=0.005). The mean titers of anti-CCP also showed a trend of decrease (114 ± 103 to 105 ± 106 units; p=0.09).

### Adverse Events

Only one patient experienced transient urticaria during the first dose of rituximab infusion. It resolved after intravenous hydrocortisone and chlorpheniramine. Drug infusion was completed without further problem. Premedication with hydrocortisone and chlorpheniramine was given to this patient before the second infusion. A mild skin reaction was noted during the infusion. This patient was known to have history of aspirin allergy and had had inadequate response to either infliximab and etanercept. No other adverse events were reported in other patients throughout the study.

## DISCUSSION

B cells may play an important role in the pathogenesis of RA. First, B cells may act as antigen presentation cells and activate T cells through the interaction between the MHC class II molecule and the T cell receptor. Second, B cells generate autoantibodies such as rheumatoid factor and anti-citrullinated cyclic peptide (anti-CCP) antibodies. These antibodies may increase the inflammatory processes in the joints by promoting immune complex formation and complement activation, and hence lead to joint erosion and damage.

The efficacy of B cell depletion in rheumatoid arthritis confirms the role of B cells in the pathogenesis of RA [6, 9]. The proportion of patients who achieved ACR responses after rituximab treatment is very similar to that of the anti-TNFα agents [1-3]. In contrast to the TNFα inhibitors, infective complications including tuberculosis and opportunistic infections are much less common with rituximab.

Our study showed that rituximab was efficacious for patients with active RA refractory to multiple DMARDs. Despite the lack of a placebo group, the improvement in our patients at the end of the study was unlikely to be spontaneous because they had active RA for at least 3 months prior to study entry. The ACR response rates achieved at week 24 were quite similar to those reported in a placebo-controlled trial of rituximab in RA [9]. In addition to the improvement in joint swelling and tenderness, there was also a significant drop in the levels of serum inflammatory markers and RF titers, which was coupled with a significant improvement in quality of life and the disability index.

Despite the fact that we did not routinely prescribe intravenous or oral corticosteroids as pre-medication before rituximab infusion, none or our patients experienced serious infusion reactions. Only one patient developed mild infusion reaction which was settled with intravenous hydrocortisone injection. Rituximab infusion was not interrupted. No infection, mild or serious, was reported throughout the 24-week follow-up in our patients. Thus, the safety profile is quite similar to that in controlled trials [9, 14] in which no significant difference in the frequency of adverse events could be demonstrated between rituximab and placebo groups of patients.

Apart from MTX and other conventional DMARDs, rituximab may also be effective in RA patients who do not respond to the anti-TNFα agents. One of our patients who had failed infliximab achieved EULAR moderate response after one single course of rituximab. An open-label uncontrolled study of rituximab (100 mg on week 1, 375 mg/m2 on week 2, 500 mg/m2 on week 3 and 4) in 13 RA patients, 6 of whom were refractory to anti-TNFα, showed that two third of patients achieved the ACR20 response at week 28 [15]. Another small uncontrolled series involving 10 patients showed that rituximab treatment (1000 mg for 2 doses 2 week apart) resulted in moderate / good DAS28 response in 80% of patients [16]. A more recent randomized controlled study called REFLEX trial (Randomized Evaluation of Long-Term Efficacy of Rituximab in RA) demonstrated that in patients who had inadequate response to one or more anti-TNFα agents, 2 infusions of 1000 mg of rituximab in addition to background MTX led to significantly higher response rates than MTX alone (EULAR moderate-to-good response 65% *vs* 22%) [14].

In summary, the current study showed that rituximab is effective in our local Chinese patients with persistently active RA despite multiple DMARD therapies including the anti-TNFα agents. Rituximab is a relatively safe option to be considered in these patients.

## Figures and Tables

**Fig. (1):**
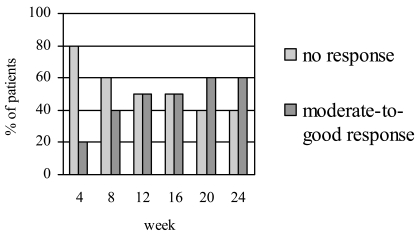
EULAR responses at different time intervals following treatment with rituximab.

**Fig. (2):**
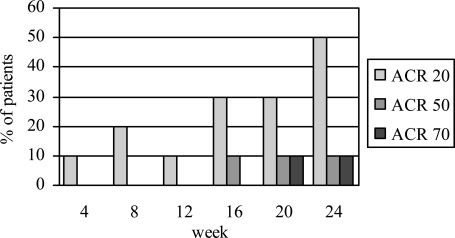
ACR20, ACR50 and ACR70 responses at different time intervals following treatment with rituximab.

**Table 1:** Clinical Characteristics of Patients at Study Entry

Age, years (median, range)	49, 36-62
Women	8 (80%)
Duration of RA, years	8, 1-19
TJC, median (IQR)	23 (17.5)
SJC, median (IQR)	11.5 (6.25)
HAQ-DI, median (IQR)	2.1 (0.67)
FACIT-F, median (IQR)	27.0 (10.8)
DAS28, median (IQR)	7.2 (1.2)
CRP, median (IQR) (mg/L)	33.1 (71.6)
ESR, median (IQR) (mm/hr)	108 (43.5)
Anti-CCP titer, median (IQR)	76 (225)
No. of patients with baseline radiographic erosions	7 (70%)
Median number of failed DMARDs	4
No. of patients who failed anti-TNFα treatment	2 (20%)

RA = rheumatoid arthritis; TJC = tender joint count; SJC = swollen joint count; HAQ = Health assessment questionnaire; FACIT-F = Functional Assessment of Chronic Illness Therapy-Fatique; DAS = disease activity score; CRP = C-reactive protein; ESR = erythrocyte sedimentation rate; CCP = citrullinated cyclic peptide; DMARD = disease modifying anti-rheumatic drugs; TNF = tumor necrosis factor.
